# Inhibition of high level *E2F* in a *RB1* proficient *MYCN* overexpressing chicken retinoblastoma model normalizes neoplastic behaviour

**DOI:** 10.1007/s13402-023-00863-0

**Published:** 2023-08-22

**Authors:** Hanzhao Zhang, Dardan Konjusha, Nima Rafati, Tatsiana Tararuk, Finn Hallböök

**Affiliations:** 1 Department of Immunology, Genetics and Pathology, Uppsala University, Uppsala, Sweden; 2 National Bioinformatics Infrastructure Sweden, Science for Life Laboratory, Uppsala University, Uppsala, Sweden; 3 Department of Medical Biochemistry and Microbiology, Uppsala University, Uppsala, Sweden; 4 Department of Immunology, Genetics and Pathology, Rudbeck Laboratory, Uppsala University, 751 85 Uppsala, Sweden

**Keywords:** Animal model, Chicken, *E2F*, Intraocular cancer, *MYCN*, *RB1* proficient, Retinoblastoma

## Abstract

**Purpose:**

Retinoblastoma, a childhood cancer, is most frequently caused by bi-allelic inactivation of *RB1* gene. However, other oncogenic mutations such as *MYCN* amplification can induce retinoblastoma with proficient *RB1*. Previously, we established *RB1*-proficient *MYCN*-overexpressing retinoblastoma models both in human organoids and chicken. Here, we investigate the regulatory events in *MYCN*-induced retinoblastoma carcinogenesis based on the model in chicken.

**Methods:**

*MYCN* transformed retinal cells in culture were obtained from *in vivo MYCN* electroporated chicken embryo retina. The expression profiles were analysed by RNA sequencing. Chemical treatments, qRT-PCR, flow cytometry, immunohisto- and immunocytochemistry and western blot were applied to study the properties and function of these cells.

**Results:**

The expression profile of *MYCN*-transformed retinal cells in culture showed cone photoreceptor progenitor signature and robustly increased levels of *E2Fs*. This expression profile was consistently observed in long-term culture. Chemical treatments confirmed *RB1* proficiency in these cells. The cells were insensitive to p53 activation but inhibition of E2f efficiently induced cell cycle arrest followed by apoptosis.

**Conclusion:**

In conclusion, with proficient *RB1*, *MYCN*-induced high level of *E2F* expression dysregulates the cell cycle and contributes to retinoblastoma carcinogenesis. The increased level of E2f renders the cells to adopt a similar mechanistic phenotype to a *RB1*-deficient tumour.

**Supplementary information:**

The online version contains supplementary material available at 10.1007/s13402-023-00863-0.

## Introduction

 Retinoblastoma is the most common intraocular childhood cancer and originates in the neural retina [1]. The majority of retinoblastomas are caused by bi-allelic *RB1* inactivation [2]. However, in a subset of cases (< 5%) *RB1* is proficient and instead other oncogenic mutations such as copy-number amplifying mutations of the *MYCN* gene (*MYCN*^*A*^) drive the carcinogenesis [3, 4]. Such retinoblastomas are formed and diagnosed earlier, have faster and more aggressive growth, are less differentiated and more prone for metastasis than *RB1*-deficient retinoblastomas without *MYCN*^*A*^ [3, 5, 6]. The *MYCN*^*A*^ amplified *RB1*-proficient retinoblastoma tumours also have distinct molecular signatures from *RB1*-deficient tumours [5]. Tumorigenic growth can be induced with high frequency when *MYCN* is over-expressed in *RB1*-proficient human retinal organoids as well as in embryonic chicken retina in vivo [7]. In both systems, the tumorigenic cells are anaplastic and express markers for active proliferation and undifferentiated cone photoreceptors (cPR) [7]. Chickens that were hatched with stable retinal *MYCN* expression, formed retinal tumours with metastatic growth that infiltrated the sclera and optic nerve and formed extraocular tumours within 6–9 weeks. The tumours had round proliferating cells, lacked Flexner-Wintersteiner rosettes but contained necrotic regions. The potential for the neoplastic growth in chicken retina is embryonic stage-dependent and feature a cell-specific resistance to apoptosis. The tumours express genes associated to the cone/horizontal cell lineage, but not to ganglion or amacrine cells [7]. Moreover, lentivirus-mediated over-expression of *MYCN* in human *RB1*-proficient foetal retina also induces tumorigenic growth resembling retinoblastoma [8]. The cellular origin lies within the undifferentiated cPR lineage. The cell-of-origin for the *RB1*-proficient tumours as shown in Blixt et al. 2022 and Singh et al. 2022 resides in the same cell type-lineage as the *RB1*-deficient tumours in the cPR lineage as shown in several independent models. However, the *MYCN*-induced *RB1*-proficient phenotype was consistently more immature and less differentiated with faster tumour progression than that of the *RB1*-deficient [5, 7–11]. The *MYCN*-transformed human foetal retina formed tumours that were not fully constrained to immature cPRs but also to a less extent developed expression of markers for e.g. ganglion cells [8]. The similarities and differences in the phenotype between the modelled variants are consistent with that seen in patients with *MYCN*^*A*^ retinoblastomas regardless of *RB1* status [3, 5].

*In vivo MYCN*-transformed chicken retinal cells grow *in vitro* as aggregates in suspension, similar to the way of established retinoblastoma lines such as WERI-RB1 and Y79. In this work we have analysed the transcriptome of established chicken retinoblastoma cells with stable *MYCN* over-expression (henceforth referred to as “DMC cells”) taken acutely (“young” DMC cells) and after culturing for more than 200 days (“old” DMC cells). Cells with over-expression of *MYCN* with the stabilizing and potentiating oncogenic mutation T58A were also analysed [12].

The retinal cell-of-origin of these transformed chick retinal cells, the cone/horizontal cell progenitor, do not exhibit developmental cell death [13, 14]. The immediate horizontal progenitor withstands DNA-damage during the terminal cell cycle and escapes both cell cycle arrest and apoptosis to continue the final cycle into differentiation [15–19]. Such natural death-resistance has been suggested to be associated with high *MDM2* expression that increases susceptibility to neoplastic transformation [15, 20]. Over-expression of *MYCN* in the progenitor cells is sufficient to drive neoplastic growth into retinoblastoma and the resistance to apoptosis in the cone/horizontal cell lineage contributes to the tumorigenic phenotype [7].

In this work we sequenced the transcriptome of *in vivo MYCN*-transformed chicken cells that have been cultured and the expression profiles contained both enriched signatures of the *RB1*-*E2F*-axis and p53 signalling pathways. The levels of *RB1* mRNA were not altered but the levels of *E2F1* and *E2F3* mRNAs were 10-fold increased. *E2F1* and *E2F3* are among the many cell-cycle regulatory genes directly or indirectly induced by Myc-proteins [21–23]. Inhibitory hyperphosphorylation of the retinoblastoma protein (Rb) in *RB1-*proficient retinoblastomas has been proposed to explain the *RB1*-deficient-like phenotype of *RB1*-proficient retinoblastomas [24]. The expression profile with high levels of *E2F mRNA* as shown in this work, opens up for a hypothesis that the increased levels of *E2F1* and *E2F3* expression may outcompete the inhibitory capacity of Rb and render a functionally *RB1* deficient phenotype. We tested this hypothesis by blocking E2fs using the small molecular inhibitor HLM006474, which normalized the cell cycle behaviour and induced cell death in the *MYCN* over-expressing cells. Moreover, blocking Cdk4/6 with Palbociclib also contributed to a partial normalization by arresting the cell cycle, supporting a partially proficient *RB1* status in the cells. The cells were arrested but did not die, and when promoting p53 using Nutlin-3a the cells did not increase apoptosis, consistent with the intrinsic p53 insensitivity of the cell-of-origin lineage. Taken together, this paper contributes to the understanding of regulatory events in *RB1*-proficient *MYCN*-overexpressing retinoblastoma. Furthermore, the study provides an explanation to how *MYCN* may promote proliferation and carcinogenesis in this retinoblastoma model of retinoblastoma tumorigenesis.

## Materials and methods

### Animals

Fertilised White Leghorn eggs (Gallus gallus; Håtunalab AB, Bro, Sweden), were incubated at 37 °C in a humidified incubator (8204/MP, Grumbach, Asslar, Germany). Embryonic age (E) was staged according to the Hamburger and Hamilton 1951 stages (st) [25]. Animal experiments were carried out in compliance with the guidelines set by the Association for Research in Vision and Ophthalmology and were approved by the regional animal ethics committee in Uppsala, Sweden (Dnr C90/16, C159/15, 5.8.18–09718/2021).

### *In ovo* electroporation of retina

Fertilized chicken eggs were windowed at st22/E3.5. Embryos were staged and electroporated with piggyBac integration expression vectors with human *MYCN*, or *MYCN-*T58A, together with green fluorescent protein (GFP) [7]. The air sac-chorioallantois boarder was marked, and a thin forceps tip was inserted in the air sac 1 mm from the mark. The inside of the egg was gently scraped in order to shift the air sac over the embryo. A piece of surgical tape was placed on the shell over the embryo and a 1 × 1 cm window was opened, producing a re-sealable “hatch”, to expose the embryo. The vitelline and chorioallantois/amniotic membranes were gently peeled open to expose the right eye of the embryo. 0.2 µl solution was injected into the subretinal space of the central retina using a capillary and mouth pipetting. 1–2 µg/µl per vector in 1x PBS with Ca^2+^ and Mg^2+^ “PBS+/+” (18912-014, Gibco, Waltham, MA, USA) and 0.02% Fast Green (F7252, Sigma-Aldrich, St. Louis, MO, USA). Fast green was added for visualization during injection. Injection was done dorsally next to the temporal posterior ciliary artery on the border of the eye and the prospective brain. The negative electrode was placed behind the central region of the eye, next to the injection, and the positive electrode was placed in front of the prospective beak. Five 50 ms pulses of 15 V with 1 s. intervals were applied using an ECM 830 Square pulse electroporator (BTX Harvard Apparatus, Holliston, MA, USA). 100 µl of Ringer’s solution was applied to the electroporated eye. The window-hatch was closed and sealed with surgical tape, and the egg incubated for continued development [7].

### Cell cultures

GFP positive (+) cells express MYCN and GFP + regions of successfully MYCN-electroporated st40/E14 retina was dissected, cells were dissociated and cultured in RPMI1640 (21875-034, Gibco) and supplemented with 10% FBS (16000044, Gibco), 1% MEM-NEAA (11140035, Gibco) and 1% pen-strep antibiotic mix (15140122, Gibco). Cells were counted by using a Countess 3 Automated Cell Counter (Invitrogen, MA, USA).

### RNA-sequencing

Cells from three acutely established primary MYCN-cell lines (denoted “young” DMC) from E14 retinas electroporated at E3.5 and cultured for 14 days, three “old” DMC cell lines cultured for > 200 days and three “old” T58A MYCN cell lines (> 200 days in culture) were triturated and used for RNA preparation. Cells from unelectroporated E14 retina were used as control and reference. RNA was extracted using the Qiagen RNeasy Micro Kit (74,004, Qiagen, MD, USA) according to the manufacturers protocol and RNA integrity was determined on a TapeStation (Agilent, CA, USA). RIN value > 8. Libraries were prepared with the TruSeq Stranded mRNA protocol using polyA-selection and sequenced on a SP1 flow cell on Illumina NoveSeq 6000 (Illumina, CA, USA).

### Sequencing data analysis

Analysis was performed at the National Bioinformatics Infrastructure Sweden. In brief, samples were checked for rRNA contamination with “bbduk” from BBMap (version 38.61) [26]. QC and alignment of the data was performed by nf-core/rnaseq pipeline (version 3.4) by adjusting the alignment parameter “alignEnsProtrude 100 ConcordantPair”. The first 10 base pairs of reads showed biased base composition and were removed. For QC, MultiQC [27] and FastQC [28] results reported by nf-core pipeline were assessed. To extract fragment counts, featurecounts (version 2.0.0) [29] was used with minimum mapping quality of 20 and a requirement of both pairs to be properly aligned on the same chromosome. Pairwise comparison was performed using edgeR [30] with pairwise comparison (exactTest). Significant differentially expressed genes (DEGs) were selected by two criteria: (1) corrected p-value for multiple testing ≤ 0.05 (FDR by the Benjamini-Hochberg procedure) and (2) ∣log2 fold-change∣ ≥ 1. 

For gene ontology (GO) terms and Kyoto Encyclopaedia of Genes and Genomes (KEGG) pathway enrichment analysis, ClusterProfiler (version 4.0.6) [31] was used on all DEGs. GO over-representation analysis was performed by “enrichGO” and GO gene set enrichment analysis was performed by “gseGO”. KEGG over-representation analysis was done with “enrichKEGG” while KEGG gene set enrichment analysis was done by “gseKEGG”. Pathway figures were obtained by Pathview (version 1.32.0). Differential expression analysis and visualization was performed in R (version 4.1.1) (R Core Team 2021) [32].

### Data availability

RNA expression data are deposited as GSE226458 at the NCBI Gene Expression Omnibus.

### Chemical treatments

1–2 × 10^6^ cells were incubated with the reagent for 24 h before analysis. The reagents targeted and inhibited MDM2 (Nutlin-3a, SML0580, Sigma-Aldrich), p53 (Pifithrin-α, P4359, Sigma-Aldrich), Cdk4/6 (Palbociclib, PZ0383, Sigma-Aldrich), E2F (HLM006474, SML1260, Sigma-Aldrich) and caused DNA damage (Cisplatin, 2251/50, Tocris, Abingdon, UK). Reagents were dissolved in DMSO, except Cisplatin that was dissolved in water, and vehicle controls consisted of an equivalent concentration of DMSO.

### Cell viability assay by Trypan Blue

1.5 × 10^6^ cells were seeded into each well of 6-well ULA plates (3471, Corning). At each time point, 200 µL cells was dissociated by gentle pipetting, filtered through 20 μm cell strainer (43-10020-60, pluriSelect, Leipzig, Germany) and mixed 1:1 with Trypan Blue (15250061, Gibco). Viability was analyzed by using a Countess 3 Automated Cell Counter (Invitrogen).

### Cell cycle analysis

Cells were centrifuged at 300 rcf for 5 min, the medium aspirated, and the cells incubated in 1X PBS-/- (14190094, Gibco). Single-cell suspensions were obtained by gentle trituration, one wash in PBS-/-, and filtration through a 20 μm cell strainer (43-10020-60, pluriSelect, Leipzig, Germany). 1 × 10^6^ cells were used per sample. The samples were washed once with PBS-/- and resuspended in 500 µl PBS-/-. To fix GFP, 500 µl of 2% PFA was added to each sample, followed by incubation at 4 °C for 1 h. The samples were centrifuged at 300 rcf for 5 min, the supernatant aspirated, and the samples washed once in PBS-/-. One ml of ice cold 70% ethanol was added dropwise under agitation and the samples were incubated at 4 °C overnight. The ethanol was aspirated following centrifugation at 1000 rcf for 5 min. The samples were resuspended in propidium iodide working solution (0.1% TritonX-100, 10 µg/ml PI; P4864, Sigma-Aldrich), and 100 µg/ml DNase-free RNase A (11119915001, Sigma-Aldrich) in PBS-/- and incubated at RT for 30 min before analysed with an easyCyte 8 Flow cytometer (Luminex Corp, the Netherlands). Aggregates or doublets were excluded from analysis. Fixed, non-electroporated retinal cells were used as a negative control to set the threshold for a positive signal. PI + or GFP + cells were gated after compensation with single-color controls, which were GFP + cells in culture and non-electroporated retinal cells with PI staining.

### Western blot analysis

Retinas and cells were homogenized in RIPA buffer (89900, Thermo Fisher Scientific) containing Halt Protease and Phosphatase Inhibitor Cocktail (78440, Thermo Fisher Scientific). The total protein concentration was measured by Dc Protein Assay kit (5000112, Bio-rad, Laboratories AB, Hercules, CA, USA). Each sample was normalized to actin level. Primary and secondary antibodies are listed in the Supplementary Table S1. The Western blot analysis was performed as previously described [33] and according to the manufacturer’s instructions.

### Quantitative reverse transcriptase PCR analysis

RNA was extracted with TRIzol (15596026, Thermo Fischer Scientific). The samples were DNase-treated (M6101, Promega, Madison, WI, USA) and cDNA was synthesized with the High-capacity RNA-to-cDNA kit (4387406, Thermo Fisher Scientific). Tests were run in triplicates using IQ™ SYBR® green Supermix (1708882, Bio-Rad) and the Ct values were normalized to β-actin and TATA box-binding protein (TBP). Control reactions containing Supermix and primers but no cDNA were run in parallel. PCR program consisted of initial denaturation step at 95 °C for 3 min, followed by 39 cycles of denaturation at 95 °C for 15 s, and annealing and extension at 60 °C for 30 s. Melt curve analysis was performed to confirm the presence of a single product. Primers were designed with Primer Express v2.0 (Applied Biosystems, Darmstadt, Germany). For primers, see Supplementary Table S2.

### Alamar blue proliferation assay

5 × 10^4^ cells were transferred to each well in 96-well ULA plates (3474, Corning, Arizona, USA). Chemical treatments were for 72 h. Alamar Blue (DAL1025, Thermo Fisher Scientific) was added 1:100 and incubated for 6 h before plate reading. The absorbance was read by ClarioStar Plate reader (BMG Labtech, Ortenberg, Germany) and the data were processed by Mars Software (BMG Labtech). Each experiment was repeated 3 times or more.

### Histo- and immunocytochemistry

Dissected chicken eyes were fixed with 4% PFA, embedded and cryo-sectioned (10 μm sections) for immunohistochemistry (IHC). For immunocytochemistry, cells were in transferred to chambered cell culture slides (354104, Corning) coated with Poly-D-Lysine (A3890401, Thermo Fisher Scientific). Suspension cells were seeded in the slides and left for sedimentation for 30 min in the incubator at 37 °C. The slides were then centrifuged at 1000 rcf for 5 min to attach the cells. Cells were washed twice with pre-warmed PBS+/+ and fixed with 4% PFA at RT for 15 min and washed twice with 1X PBS. The permeabilization was performed by incubation with 0.2% TritonX-100 in PBS for 20 min at room temperature and washed twice with 1X PBS. After permeabilization, the blocking and antibody incubation were the same as immunohistochemistry. TUNEL staining was performed with Click-iT TUNEL assay according to manufacturer’s instruction (C10619, Thermo Fisher Scientific). For antibodies, see Supplementary Table S1.

### Microscopy and Image analysis

Fluorescence micrographs were captured using a Zeiss Imager Z2 microscope (Carl Zeiss Microscopy GmbH, Jena, Germany). Quantification of TUNEL stained cells was assisted by the software Fiji-ImageJ2. Contrast of fluorescence images was enhanced at the microscope using the Zeiss capture and image analysis software (ZEN).

### Data analysis

Data were analysed with one-way ANOVA followed by Tukey’s multiple comparison post-hoc test or Student’s t test using GraphPad Prism (GraphPad Software Inc. San Diego, CA, USA) and statistical significance was set to p < 0.05. Statistical analysis and number of replicates are presented in the figure legends.

## Results

### *MYCN*-transformed retinal cells grow in vitro in suspension and are *RB1*-proficient

**Fig. 1:**
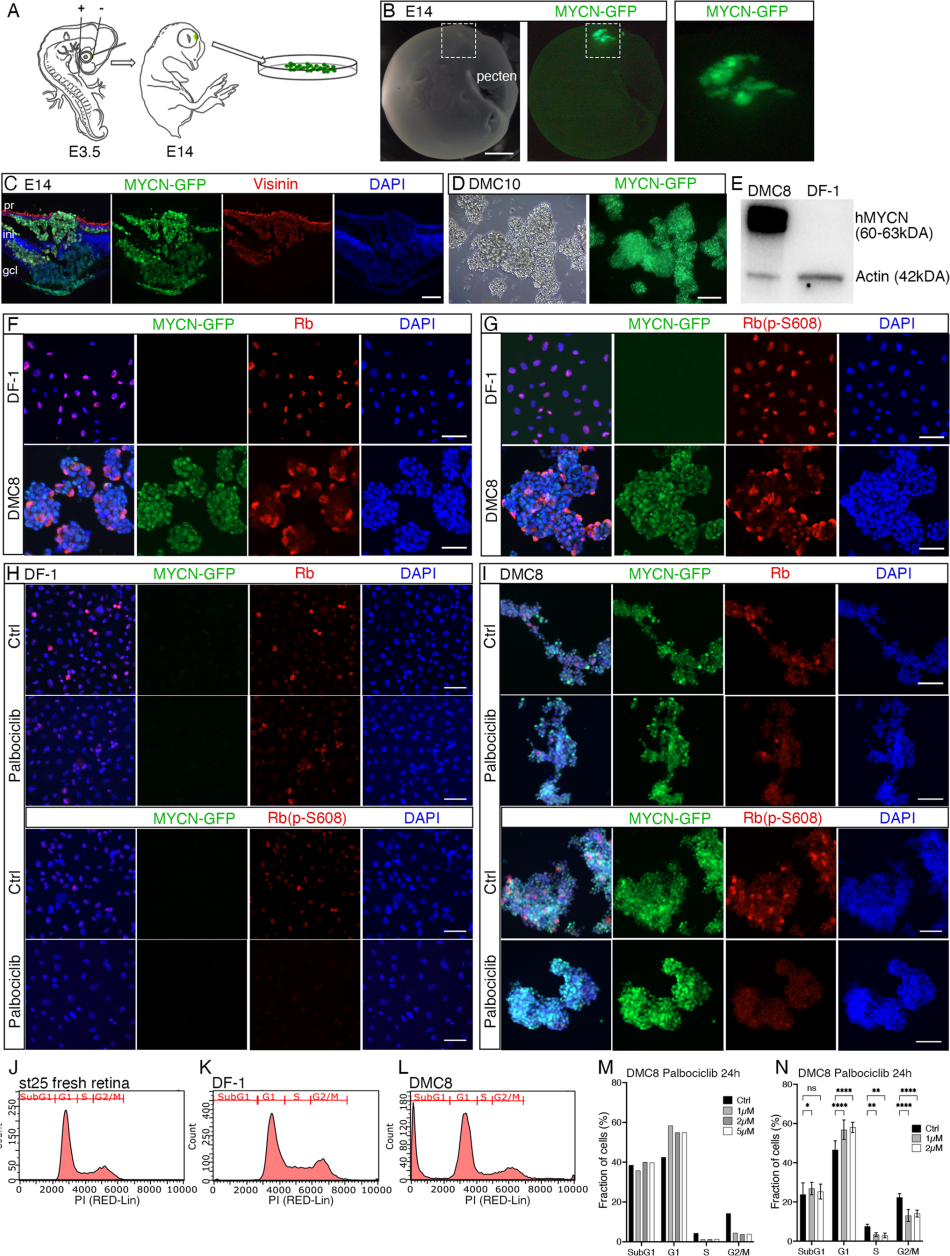
Establishment and cellular properties of *MYCN*-overexpressing cell lines with proficient *RB1.* Fluorescence micrographs of E14 (st40) electroporated chicken eye and DMC cell lines. Bar graph and histogram of cell cycle analysis. **A** Schematic illustration of subretinal injection and electroporation of E3.5 (st22) embryo. **B** Representative micrographs of *MYCN*-electroporated E14 retina. Dashed box is magnified in the right panel. **C** Immunohistochemistry micrographs of *MYCN* electroporated E14 retina stained for GFP and Visinin. **D** Representative micrographs of live DMC cells in culture with brightfield and GFP. **E** Western blot of human *MYCN* level in DMC cells and DF-1 control cells. **F** Immunocytochemistry of DMC and DF-1 cells stained for Rb and GFP. **G** Immunocytochemistry of DMC and DF-1 cells stained for phosphorylated Rb (P-S608) and GFP. **H-I** Immunocytochemistry of DF-1 and DMC cells stained for Rb, phosphorylated Rb (P-S608) and GFP after 2 µM Palbociclib treatment. **J-L** Representative images of cell cycle analysis from flow cytometry of st25 (E4.5) fresh retina, DF-1 and DMC cells. **M** Bar graph of percentage of DMC cells in cell cycle phases after different doses of Palbociclib treatment. **N** Bar graph of percentage of DMC cells in cell cycle phases after 1 µM and 2 µM Palbociclib treatment. Mean ± SD, ****p < 0.0001, ***p < 0.001, **p < 0.01, *p < 0.05, **N***n* = 3. **A** E; embryonic day, **C** pr; photoreceptor layer, inl; inner nuclear layer, gcl; ganglion cell layer, **J** st; Hamburger &Hamilton developmental stage. Scale bars in **B**, 500 μm; in **C**, 200 μm; in **D**, 300 μm; in **F-I**, 50 μm

*MYCN* over-expression was achieved after *in ovo* electroporation of normal E3.5 embryonic chicken *RB1-*proficient retina (Fig. 1A) using a piggyBac integrating *MYCN*-expression vector. Stable over-expression of human *MYCN* and GFP cDNA sequences was established using a polycistronic expression unit driven by the chicken actin promoter (CAG) [7]. Electroporated retinas were taken at E14 and large clusters of GFP positive (+) and Visinin immunoreactive (IR) cells disrupted normal retina morphology (Fig. 1B and C). Visinin is a recoverin homologue [34] and a specific cell type marker of cPR progenitors [35], indicating a cPR fate of these cells. The GFP + regions of E14 retinas were dissected, dissociated and transferred to cell cultures. The *MYCN*-transformed GFP + cells survived and proliferated in vitro, whilst GFP-negative cells all died within two weeks. The GFP + cells, denoted DMC, grew in suspension and in clusters similarly to previously established retinoblastoma cell lines and in the same type of medium (WERI and Y79 [36]) (Fig. 1D). Western blot analysis showed that the transgene *MYCN* protein was robustly over-expressed in the DMC cells, compared to chicken fibroblast cells (DF-1 cells), which were used as control cells (Fig. 1E).

In order to verify the RB1 expression and proficiency, we used IHC to analyse total Rb and Rb phosphorylated on S608, Rb(P-S608) in DMC cells and DF-1 cells. Phosphorylation of the S608 inhibits E2F binding and blocks E2F activity [37]. GFP + DMC cells were immunoreactive (IR) for both Rb and Rb(P-S608) to a similar degree as the control DF-1 cells (Fig. 1F and G) indicating that *RB1* was expressed, could be phosphorylated and that the regulatory system of RB1 was active. The main regulatory enzymes for Rb phosphorylation are Cdk4/6 [38] and 2µM of the Cdk4/6 inhibitor Palbociclib abolished Rb(P-S608) IR in both DF-1 and DMC cells (Fig. 1H and I), indicating that the CDK4/6-RB1 regulatory system is active in the DMC cells. We analysed the cell cycle of DMC cells and as control we used dissociated normal st25/E4.5 retinal cells and DF-1 cells. The st25/E4.5 retina has proliferating retinal cells and normal E14 cells are mainly post mitotic. Analysing the relative proportion of cells in the cell cycle phases using the DNA content per cell showed that DMC cells had a distorted cell-cycle profile compared to the st25 retina and DF-1 cell-controls, with a conspicuous sub-G1 peak representative for on-going apoptosis (Fig. 1J-L). Cell death was observed in the DMC lines, consistent with the cell cycle profile, but not to an extent that the net positive growth was affected. We analysed the dose response of Palbociclib on the cell cycle in DMC cells (1, 2 and 5 µM, Fig. 1M) and treatment with 1 and 2 µM Palbociclib resulted in a significant lengthening of the G1-phase and a shorter G2/M-phase, indicative of a partial G1-phase cell cycle arrest (Fig. 1N). Increased G1-phase arrest by Palbociclib is consistent with proficient Rb as shown after treatment of breast cancer cells [39]. The effects corroborated that *RB1* in DMC cells is expressed and has at least a partial capacity to regulate the cell cycle.

### RNA sequencing of *MYCN*-expressing DMC cells

**Fig. 2:**
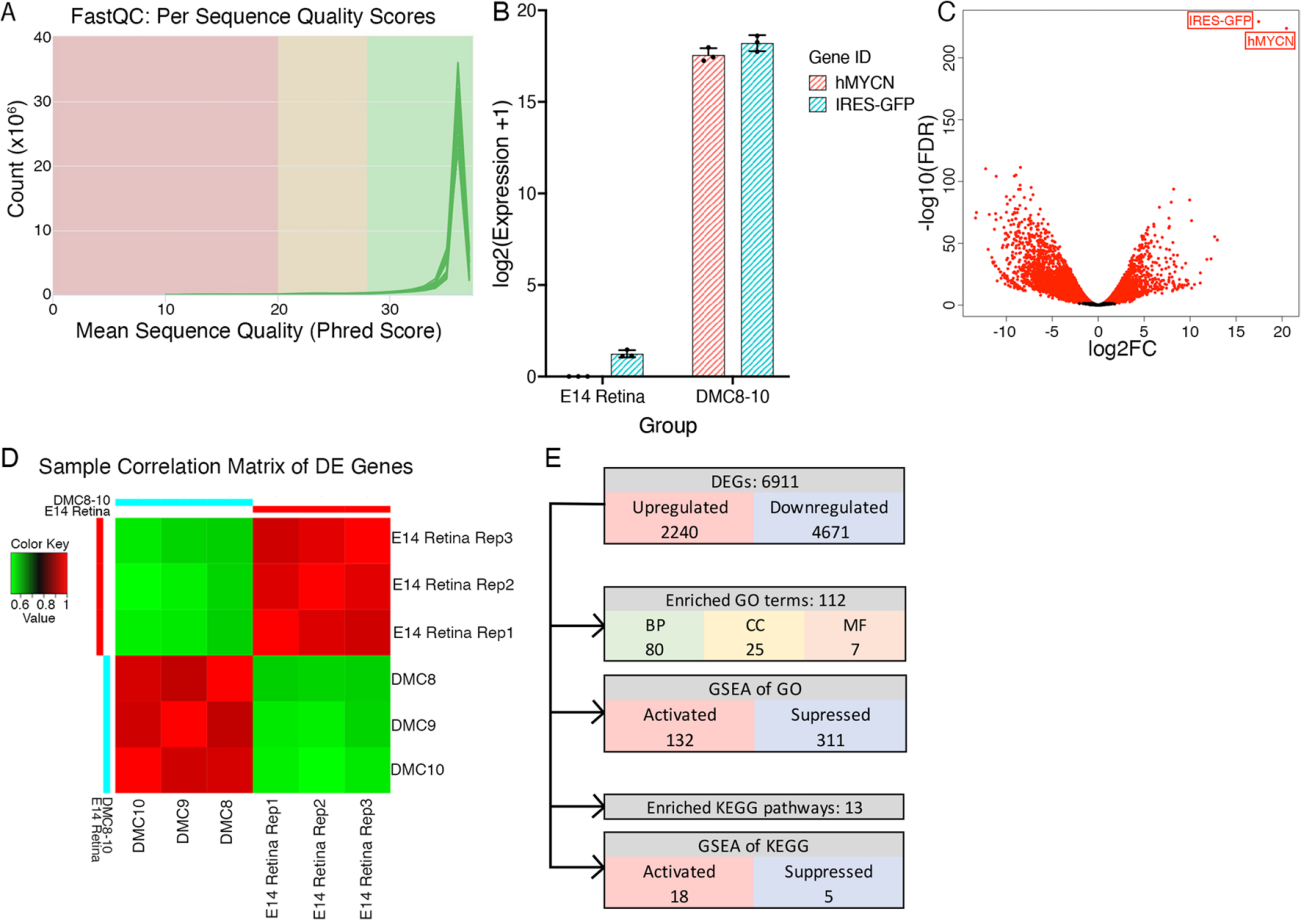
Bulk RNA sequencing of young DMC cells. Results from Bulk RNA sequencing of DMC cells with unelectroporated E14 retina as control. **A** Plot with per sequence quality scores of all replicates of DMC cells. **B** Expression level of human *MYCN* (*hMYCN*) and GFP (IRES2-GFP) in DMC cells and control E14 unelectroporated retina. **C** Volcano plot of annotated genes in DMC cells and E14 control retinas. Differentially expressed (DE) genes are shown in red (FDR < 0.05, log2FC > 1). IRES-GFP and *hMYCN* were marked out. **D** Sample correlation matrix based on DE genes of DMC and control unelectroporated E14 retinas. **E** Overall results of differential expression analysis, GO enrichment analysis, gene set enrichment analysis (GSEA) of GO, KEGG pathway enrichment analysis and GSEA of KEGG pathways

We performed bulk RNA sequencing on RNA prepared from three acutely established cell lines from MYCN-transformed E14 retina and three normal E14 chicken retina samples, used as matching controls. The acutely established cells, also denoted “young” DMC cells, had been in culture for 2 weeks until all GFP negative cells were gone. All samples passed the quality control of sequencing results. The per sequence quality scores were over 35 (Fig. 2A) and h*MYCN* and GFP sequences was only detected in the DMC cells (Fig. 2B and C). Expression analysis identified 6911 differentially expressed (DE) genes, including 4671 down- and 2240 up-regulated in DMC cells compared to E14 retina (Fig. 2C, Supplementary Table S3). The sample correlation matrix showed expression difference between the groups and consistent expression pattern among the replicates within each group (Fig. 2D). Over-representation of GO and KEGG pathways gave 112 enriched GO terms and 13 enriched KEGG pathways (Fig. 2E).

### *MYCN*-expressing DMC cells exhibit a proliferating cell death-deregulated cone photoreceptor progenitor phenotype

**Fig. 3:**
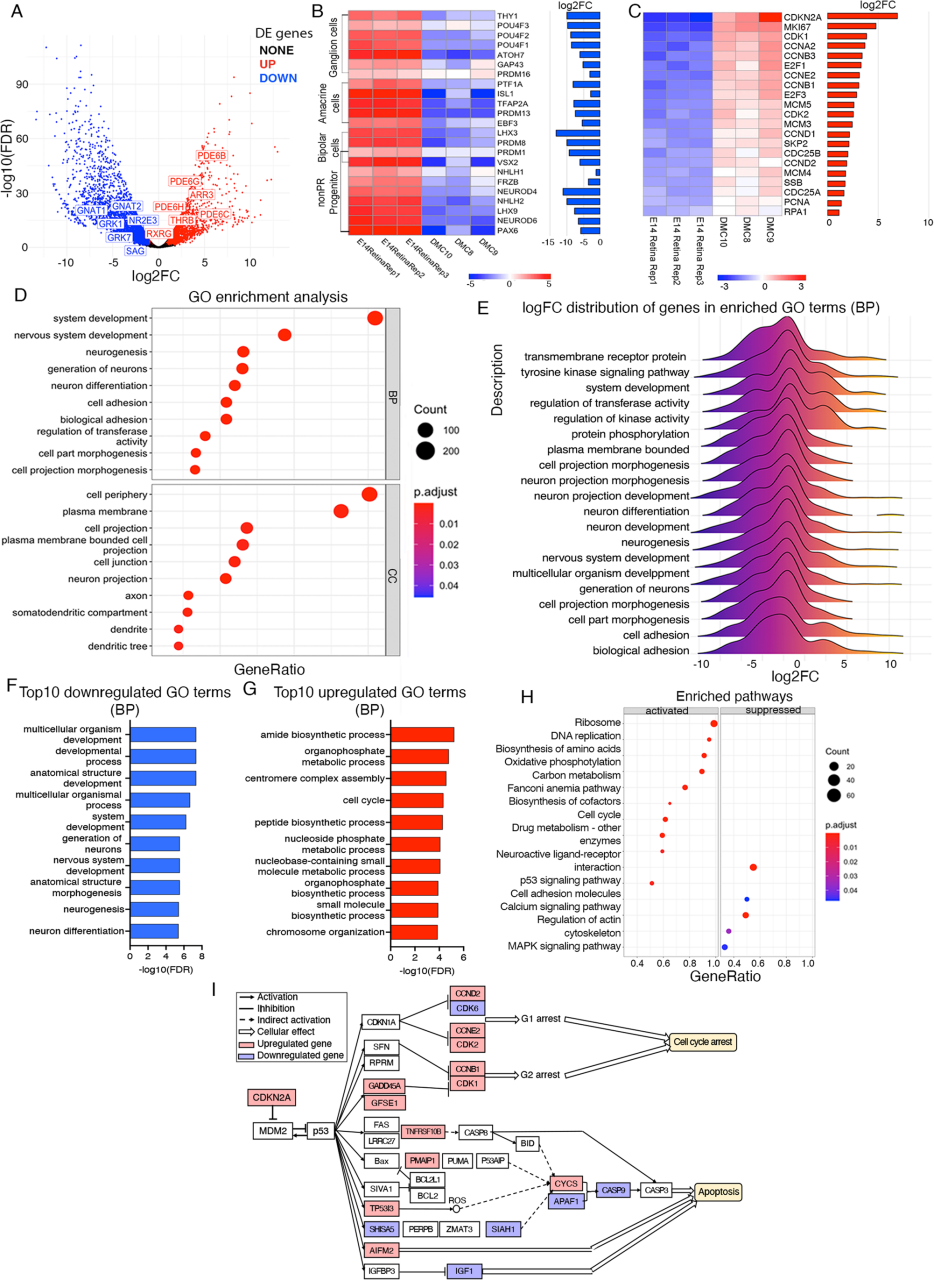
Differential expression analysis and analysis based on differentially expressed (DE) genes. Results from differential expression analysis of comparison between DMC and E14 retina and subsequent over-representation analysis and gene set enrichment analysis (GSEA) of gene ontology (GO) and KEGG pathways. **A** Volcano plot of annotated genes, with downregulated genes shown in blue and upregulated genes in red. Markers of photoreceptors were marked out. **B** Heatmap of expression of markers for ganglion cells, amacrine cells and progenitors in non-PR fate. **C** Heatmap of expression of markers for cell cycle and proliferation. **D** Top 10 enriched GO terms in BP and CC from over-representation analysis. **E** Plot with distribution of lof2FC in top 20 enriched GO terms in BP. **F-G** Top 10 downregulated and upregulated GO terms in BP from GSEA. **H** Top 15 enriched pathways of GSEA of KEGG pathways. **I** Pathview of cell cycle and apoptosis related genes in the p53 signaling pathway, with upregulated genes in red and downregulated genes in blue. PR; photoreceptor, FDR; false discovery rate adjusted *p* value, log2FC log2 fold change, BP; biological process

The expression profiles of DMC cells have markers for cPR, among which, some were differentially expressed (Fig. 3A). The expression of key components of the cone and rod phototransduction pathways was compiled in Supplementary Table S4 including opsins; OPNs, transducins; GN, phosphodiesterases; PDEs, cyclic nucleotide-gated ion channels; CNGs, guanylyl-cyclases; GDs, G-coupled receptor kinases; GRKs, arrestins, recoverins and guanylyl cyclase-activating proteins; GUCAs [40, 41]. Because E14 retina contains developing cones, differential expression between DMC cells and E14 retina was not a criterion for inclusion in the compilation, but several of the genes were differentially expressed indicating in many cases that their expression was enriched in the DMC cells. All major components in the phototransduction pathway, which also have annotated chicken orthologs, were found in the expression data and several of the cPR enzymes were upregulated (*OPN1LW*, *PDE6C, PDE6H*, *ARR3*, *GUCA1A, GUCA1B*) and some of the rod genes were downregulated (*SAG, GRK1, GNB1, NR2E3*), a pattern that is consistent with the hypothesized cPR phenotype of these transformed cells. However, not all the regulatory genes followed this pattern. The a-subunit of cone transducin (*GNAT2*) and the cone G-protein-coupled receptor kinase (*GRK7*) were downregulated compared to E14 retina (although from high levels), while the rod-specific phosphodiesterases *PDE6B* and *PDE6G* were upregulated (Fig. 3A, Supplementary Table S4). Genes associated with cPR progenitors, Thyroid receptor b (*THRB*) and RXR-g (*RXRG*), were enriched in the DMC cells compared to retina while genes related to rod development were downregulated (*NR2E3*) (Fig. 3A). Markers for ganglion cells, amacrine cells, bipolar cells and progenitors for non-PR fate were also downregulated (Fig. 3B). These results show that the acutely isolated DMC cells have an expression profile of a cPR progenitor phenotype.

The cells proliferated, which is in contrast to most of the E14 retinal cells which are post-mitotic, and thus we examined genes related to proliferation. Genes related to the cell cycle were upregulated and the high expression of *MKI67* and *PCNA* are indicative of proliferation. Cyclins (*CCNA2, CCNB3, CCNE2, CCNB1, CCND1, CCND2*) and cyclin-dependent kinases (*CDK1, CDK2*) as well as the cell cycle regulators *CDKN2A, SKP2, CDC25A, CDC25B*, and *AURKA* were found to be among the top upregulated genes in DMC compared to E14 retina control (Fig. 3C, Supplmentary Tables S3 and S5). *E2F1*, *E2F3, E2F6* and *E2F8* were upregulated while *RB1* showed no significant expression difference. Because of functional association between *E2F*s and *RB1* and the high expression levels of *E2F*s we further explored these genes. Several of the genes that regulate the cell cycle and proliferation are upregulated and are also known regulatory targets of the Myc-transcription factors [23]. A compilation of the expression of Myc-regulated genes described in Bretones et al. 2015 in the “young” DMC cells is shown in Supplementary Table S5.

Next, we applied analysis of GO and KEGG to link DE genes to biological activities: biological processes (BP) and cellular components (CC). The top enriched GO terms in BP included system and neuronal development or differentiation and were all downregulated while the upregulated GO terms were associated with cell cycle and proliferation (Fig. 3D-G, Supplementary Fig. S1). These results imply a less differentiated and highly proliferative phenotype of the DMC cells compared to E14 retina. Such phenotype is consistent with the phenotype of retinoblastoma with *MYCN* amplifications [3, 4, 42, 43].

The top 15 enriched activated and suppressed KEGG pathways confirmed that the DMC cells expressed genes associated with increased proliferation with active cell cycle and DNA replication (Fig. 3H). The p53 signalling pathway was among the identified pathways with both up and down regulated components, indicating that MYCN cells are differently regulated compared to E14 retina (Fig. 3I). The p53 induced genes *TP53I11, TP53INP1, TP53IBP1* and *TP53IBP2* that are all down regulated while *MDM2* as well as *MDM4* were not changed (Supplementary Table S3). One of the top upregulated DE gene is *CDKN2A* (Fig. 3C), which is involved in cell cycle, p53 regulatory and senescence pathways, although the role for *CDKN2A/B* in chicken is not identical to the one in humans and is less well studied [44]. The identified pathways are consistent with the observed phenotype of the DMC cells with ongoing proliferation and dysregulated apoptosis.

We also compared the expression profile of data sets of two patient-derived *MYCN*^*A*^ tumours, one *RB1* proficient; GSE161449 [45] and one *RB1* deficient; OEP 000103 [46]. Comparison showed that the top enriched GO-terms partially overlapped (Supplementary Fig. S2).

### Effects of p53 modulators Nutlin-3a and Pifithrin-α on the cell cycle of DMC cells

**Fig. 4:**
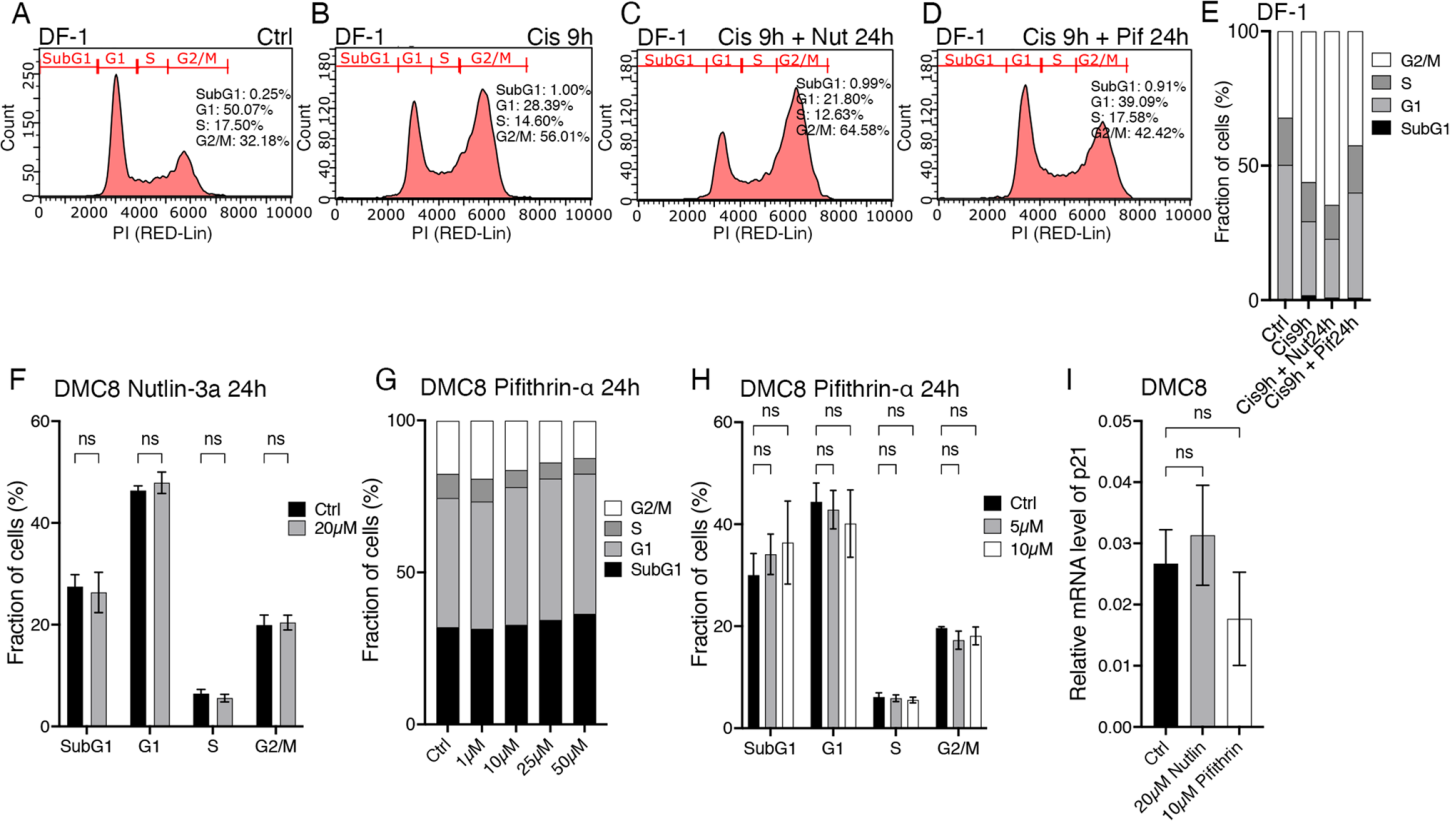
Effects of p53 modulators on the cell cycle of DMC cells. DF-1 cells were used as control and for testing drugs. Cell cycle analysis was performed by flow cytometry and p21/CDKN1A mRNA levels were determined by qRT-PCR. **A-D** Representative images with cell cycle phases from flow cytometry of cell cycle analysis. DF-1 cells were treated with 5 µM Cisplatin, together with 20 µM Nutlin-3a or 10 µM Pifithrin-α. **E** Stacked bar graph of cell cycle phases of DF-1 cells from A-D. **F** Bar graph of cell cycle phases of DMC cells after treatment of 20 µM Nutlin-3a for 24 h. **G** Stacked bar graph of cell cycle phases of DMC cells after Pifithrin-α treatment for 24 h. **H** Bar graph of cell cycle phases of DMC cells after treatment of 5 and 10 µM of Pifithrin-α. **I** Bar graph with relative mRNA levels of p21 in DMC cells after treatment with either 20 µM Nutlin-3a or 10 µM Pifithrin-α for 24 h. Mean ± SD, ns; not significant (*p* > 0.05), ANOVA in **F** *n* = 3, **H** *n* = 4, **I** *n* = 6. Ctrl; control, Cis; Cisplatin, Nut; Nutlin-3a, Pif; Pifithrin-α

The developmental retinal cell lineage from which the chicken retinoblastoma DMC cells are derived [7], exhibit p53 insensitivity [16, 17]. This together with the identified p53 KEGG pathway (Fig. 3I) prompted us to study the cell cycle and cell death regulation of these cells. We used the Mdm2 inhibitor Nutlin-3a and p53 inhibitor Pifithrin-α to modulate p53. Nutlin-3a promotes p53 activity by blocking Mdm2, a negative regulator of p53 [47], and Pifithrin-α is a widely used p53 inhibitor [48], although its specificity and mode of action have been challenged [49]. We treated the control DF-1 cells with Cisplatin, which inflicts DNA damage and induces cell cycle arrest in a p53-dependent manner in normal cells [50]. The DF-1 cells were cell cycle arrested in G2-M phase compared to untreated controls (Fig. 4A, B and E). Nutlin-3a treatment augmented the cisplatin-induced arrest (Fig. 4C and E) while Pifithrin-α had a normalizing effect and released the arrest (Fig. 4D and E).

Having identified the expected cellular response in p53-functional cells, we treated the “young” DMC cells with 20 µM Nutlin-3a (Fig. 4F and I) or Pifithrin-α (Fig. 4H-I) but no obvious effects on the cell cycle were observed. For Pifithrin-α treatment, a dose-response study was performed to decide an efficient dose (Fig. 4G). 5 µM and 10 µM Pifithrin-α were then used but no significant effects could be seen on the DMC cells (Fig. 4H). We analysed the p21 (*CDKN1A*) mRNA levels by qRT-PCR. p21 is downstream, positively regulated by p53 but no significant change on CDKN1A/p21 mRNA levels by Nutlin-3a or Pifithrin-α was seen (Fig. 4I). We concluded that manipulation of p53 activity neither induced changes in the cell cycle nor on p21 mRNA levels in the DMC cells. Although several genes in p53 signalling pathway were identified as altered among the DE genes (Supplementary Table S5), the DMC cells did not seem to respond to direct manipulation of p53 activity.

### Effects of E2f-inhibitor HLM006474 on cell cycle and apoptosis in DMC cells

**Fig. 5:**
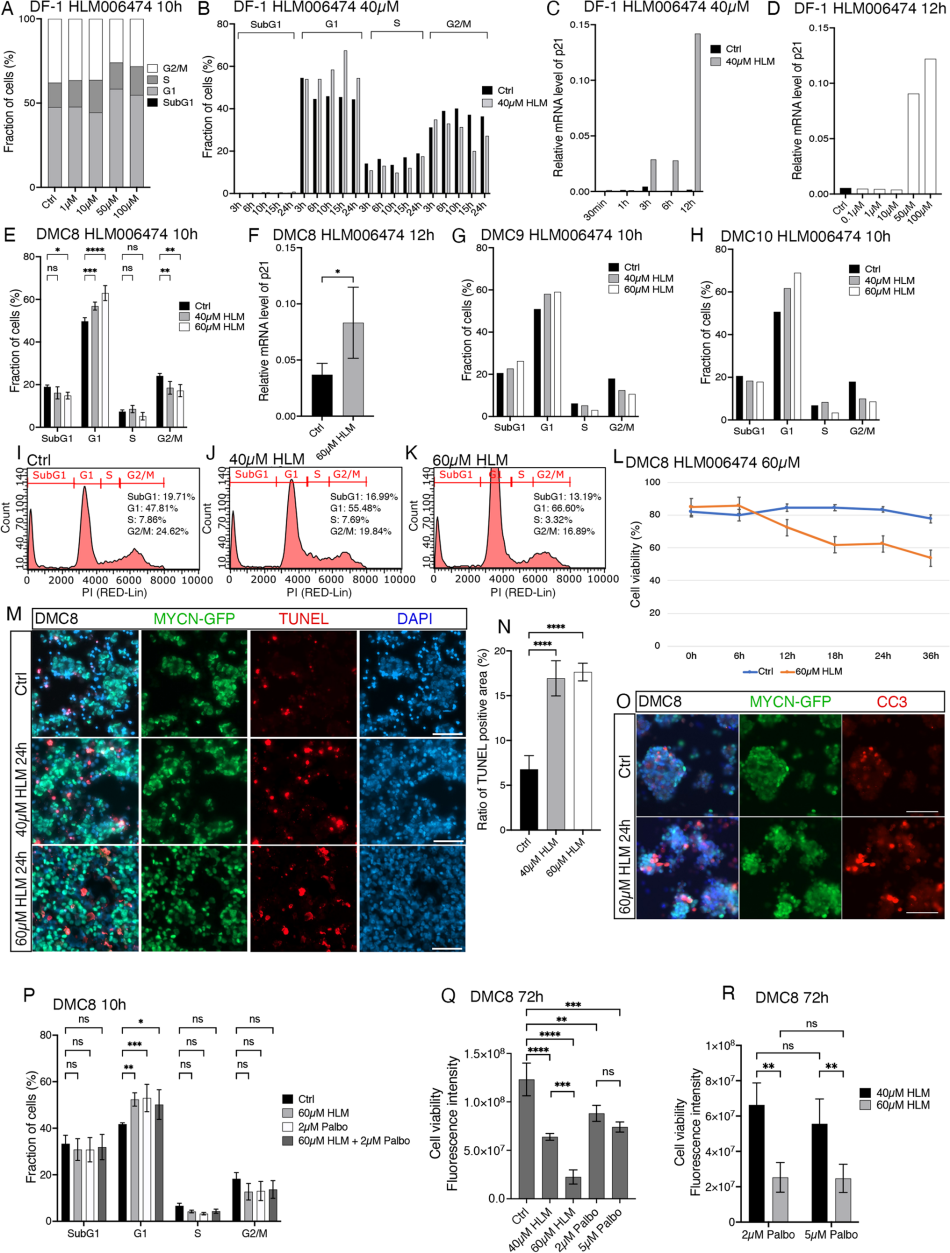
Effects of E2f inhibitor HLM006474 on “young” DMC cells. E2f was chemically inhibited by HLM006474 treatment on DF-1 and DMC cells. Cell cycle and cell viability were examined after treatment. Expression level of p21/CDKN1A was also measured. **A** Stacked bar graph of cell cycle phases of DF-1 cells after 10 h HLM006474 treatment of different doses. **B** Bar graph of cell cycle phases of DF-1 cells after 40 µM HLM006474 treatment at different time points. **C** Bar graph of relative mRNA level of p21 in DF-1 cells after 40 µM HLM006474 treatment of different treatment times. **D** Bar graph of relative mRNA level of p21 in DF-1 cells after 12 h HLM006474 treatment of different doses. **E** Bar graph of cell cycle phases after 10 h treatment of 40 and 60 µM HLM006474 on DMC8 cells. **F** Bar graph of relative mRNA level of p21 after 12 h 60 µM HLM006474 treatment on DMC8 cells. **G-H** Bar graphs of cell cycle phases after 10 h treatment of 40 or 60 µM HLM006474 on DMC9 and DMC10 cells. **I-K** Representative images of cell cycle analysis from flow cytometry of control DMC cells (Ctrl), 10 h 40 µM and 10 h 60 µM HLM006474 treated DMC cells. **L** Cell viability over time following 60 µM HLM006474 treatment on DMC8 cells, analyzed by Trypan blue assay. **M** Representative fluorescence micrographs of TUNEL staining of control DMC8 cells (Ctrl), 24 h 40 µM HLM006474 treated and 24 h 60 µM HLM006474 treated DMC8 cells. **N** Bar graph of the ratio of positive area of TUNEL over positive area of DAPI, measured by ImageJ. **O** Representative fluorescence micrographs of cleaved caspase-3 IR of 24 h 60 µM HLM006474 treated DMC8 cells. **P** Bar graph of cell cycle phases after 10 h 2 µM Palbociclib treatment and/or 60 µM HLM006474 treatment on DMC8 cells. **Q** Bar graph of cell viability measured by fluorescence intensity after different doses of HLM006474 and Palbociclib treatment on DMC8 cells for 72 h, analyzed by Alamar Blue assay. **R** Bar graph of cell viability measured by fluorescence intensity after 72 h 40 µM and 60 µM HLM006474 treatment, together with 2 µM or 5 µM Palbociclib treatment on DMC8 cells, analyzed by Alamar Blue assay. Mean ± SD, ****p < 0.0001, ***p < 0.001, **p < 0.01, *p < 0.05, ns not significant (*p* > 0.05), ANOVA in **E**, **F, L, N and P** *n* = 4, in **Q and R** *n* = 3. HLM; HLM006474, h; hours, Palbo; Palbociclib, CC3; cleaved caspase-3. Scale bars in **M and O** is 50 μm

Constitutively active *E2Fs* during the cell cycle as a result of *RB1* inactivating mutations is a major driving force in retinoblastoma carcinogenesis [2]. *E2F1* and *E2F3* mRNA levels were robustly elevated in the DMC cells when compared to E14 retina. The *RB1* levels were however not elevated (Supplementary Fig. S3). We hypothesized that the elevated *E2F* levels could override the inhibitory regulatory function that Rb has on E2fs. Increased *E2F1* and *E2F3* and unaltered *RB1* expression was confirmed using qRT-PCR (Supplementary Fig. S3). However, because none of the tested anti-E2f-antibodies worked on the chicken material in our hands for IHC or western blot analysis we could not confirm the result on protein level. To test whether the high levels of *E2F* expression contributed to the uncontrolled growth of DMC cells, we treated DMC cells with the E2f inhibitor HLM006474. HLM006474 is a small molecule inhibitor of the DNA binding abilities of E2fs [51, 52]. DF-1 cells were used as control and effects of the E2f inhibitor was assessed by studying the cell cycle and proliferation. We assessed the dose-response and time-line effects on DF-1 cells and 40 µM HLM006474 arrested DF-1 cells in G1 with a robust effect after 10–15 h of treatment (Fig. 5A and B). Treatment for 24 h or more, or with 100 µM or more reduced the cell viability to the point where cell cycle analysis of DF-1 cells was not possible. We also analysed the *CDKN1A* mRNA levels in treated DF-1 cells and found a robust increase after 10 h with 50 µM E2f inhibitor (Fig. 5C and D), results that were consistent with the cell cycle analysis.

We treated DMC cells with 40 or 60 µM HLM006474 during 10–12 h. Both concentrations of HLM006474 caused cell cycle arrest compared to control (Fig. 5E). Moreover, *CDKN1A/p21* mRNA levels were significantly increased (Fig. 5F). The effect of HLM006474 on the cell cycle of DMC cells (Fig. 5I-J) was confirmed in two additional independently established DMC cell lines from separately electroporated retinas (DMC9 and DMC10). The effects on the cell cycle could be replicated in all three cells (Fig. 5G and H).

Sustained treatment with HLM006474 for 36 h caused cell death as shown both using Trypan blue exclusion assay, TUNEL staining and cleaved Caspase-3 IR (Fig. 5L-N) implying that the death was by apoptosis. TUNEL staining was significantly higher with 40 and 60 µM HLM006474 compared to control (Fig. 5N). HLM006474 treatment also induced the cleavage of caspase-3 as shown by cleaved caspase-3 IR, which is a marker of apoptosis (Fig. 5O). This result indicated that the E2f inhibitor induced cell cycle arrest followed by apoptosis, a result that gave support to the hypothesis that increased levels of *E2F*s contributes to the neoplastic phenotype in this *RB1* proficient retinoblastoma model.

Next, we investigated whether the Cdk4/6 blocker Palbociclib interacted with the effect of the E2f inhibitor. Both Palbociclib and HLM006474 by itself induced G1-phase arrest after 10-hour treatment of DMC cells and no additive effects were seen by combining the reagents (Fig. 5P). However, when the treatment was prolonged till 72 h and proliferation/viability was studied, the effect of HLM006474 was larger than that of Palbociclib (Fig. 5Q). A significant increase between 40 and 60 µM HLM006474 but not between 2 and 5 µM Palbociclib was seen (Fig. 5Q), indicating that the effect of Palbociclib was saturated and could not optimally block proliferation, while that of HLM006474 was not saturated at the tested concentrations. When combined, the effect on proliferation/viability was larger with increasing HLM006474 concentrations but not with increasing Palbociclib concentrations (Fig. 5R), supporting the hypothesis that regulation of Rb by Cdk4/6 cannot fully control proliferation and that HLM006474 acts downstream on E2fs in these cells. This is also consistent with that although the combination of HLM006474 and Palbociclib did not have any additive or synergistic effects on DMC cells, HLM006474 had a more potent effect than Palbociclib.

### DMC cells cultured for > 200 days retain the photoreceptor progenitor phenotype

**Fig. 6:**
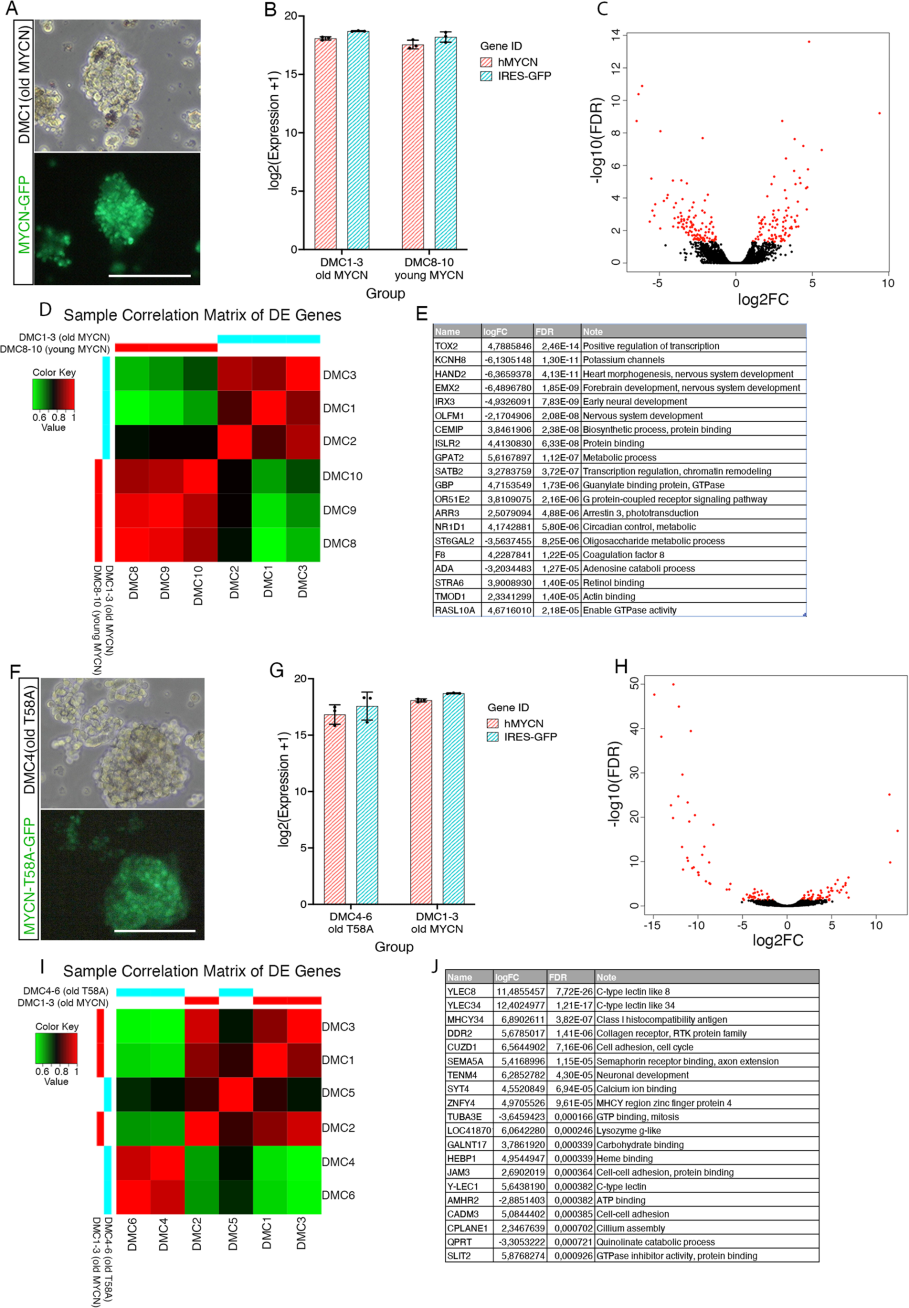
RNA sequencing analyses of old and T58A DMC cell lines. RNA sequencing results from comparisons: DMC8-10 vs. DMC1-3, DMC4-6 vs. DMC1-3. DMC1-3 were with wild type MYCN variants, over 200 days in culture (“old” DMC). DMC4-6 were with MYCN-T58A variants, over 200 days in culture (*MYCN*-T58A DMC). **A** Brightfield and GFP fluorescence micrographs of DMC1 in culture. **B** Expression level of human MYCN (*hMYCN*) and GFP (IRES2-GFP) in DMC1-3 and DMC8-10. **C** Volcano plot of annotated genes in the comparison of DMC8-10 to DMC1-3. Differentially expressed (DE) genes were shown in red (FDR < 0.05, log2FC > 1). **D** Sample correlation matrix based on DE genes of comparison of DMC8-10 to DMC1-3. **E** List of top 20 DE genes according to FDR in the comparison of DMC8-10 to DMC1-3. **F** Brightfield and GFP fluorescence micrographs of DMC4 in culture. **G** Expression level of human MYCN (*hMYCN*) and GFP (IRES2-GFP) in DMC4-6 and DMC1-3. **H** Volcano plot of annotated genes in the comparison of DMC4-6 to DMC1-3. Differentially expressed (DE) genes were shown in red (FDR < 0.05, log2FC > 1). **I** Sample correlation matrix based on DE genes of comparison of DMC4-6 to DMC1-3. **J** List of top 20 DE genes according to FDR in the comparison of DMC4-6 to DMC1-3. FDR; false discovery rate adjusted *p* value, log2FC log2 fold change. Scale bars in **A** and **F** is 300 μm

DMC 8, 9 and 10 were sequenced and analysed as acutely established MYCN expressing cells (“young” DMCs). We compared the expression profile of “young” DMCs with three established lines DMC 1, 2 and 3 (“old” DMCs), which had been in culture for more than 200 days. The “young” and “old” DMC cells had similar morphologies and grew in suspension in clusters (Fig. 6A). The results from RNA sequencing confirmed the high expression of human MYCN and GFP (Fig. 6B). The expression of key components of the cone and rod phototransduction pathways was similar in the “young” and “old” DMCs with expression of all major pathway components (Supplementary Table S6).

Differential gene expression analysis of “young” versus “old” DMCs identified in total only 211 DE genes: 95 upregulated and 116 downregulated (Fig. 6C). The sample correlation matrix based on DE genes showed that one of the old cells, DMC2 clustered with the group of “young” replicates. Over-representation and gene GSEA of GO and KEGG pathways did not give any hits. This is consistent with that the “young” and “old” cells are similar and that the cPR lineage phenotype is maintained over extended time in culture (Fig. 6D). Among the top 20 DE genes, many upregulated genes in the “young” DMC cells were involved in metabolic process or transcription (*TOX2, CEMIP* and *GPAT2*) while the downregulated genes were involved in development or neuron differentiation (*HAND2, EMX2, IRX3* and OLFM1) (Fig. 6E). *ARR3*, in the phototransduction pathway was DE and had lower expression level in the “old” DMC cells. *STRA1* (receptor for retinol binding protein) and *ABCA4* (retinoid transporter/flipase) were also differentially expressed and had lower expression level in the “old” cells. Moreover, although not statistically supported, there was a trend that other phototransduction genes were less upregulated in the “old” cells than in the “young” DMC cells compared to E14 retina (Supplementary Table S6).

### Expression profiles of “old” MYCN DMC and MYCN-T58A DMC cells are to a large degree similar

The “old” DMC cells, *MYCN*, (DMC 1–3 > 200 days in culture), and *MYCN-*T58A cells, (DMC4-6, > 200 days in culture) were compared. *MYCN-*T58A is *MYCN* with the oncogenic mutation in T58 that increases the stability of the Myc protein and produces robust tumour growth. The “old” DMC and the *MYCN*-T58A DMC cells had similar morphology, grew in suspension in clusters and expressed GFP (Fig. 6F). The results from RNA sequencing confirmed equally high expression of hMYCN and GFP in the ”old” DMC and the *MYCN*-T58A cells (Fig. 6G). The expression of key components of the cone and rod phototransduction pathways in the *MYCN*-T58A cells was very similar to both the “young” and “old” DMCs with expression of all major phototransduction pathway components, although at different levels (Supplementary Table S7).

Analysis of differential gene expression identified 136 DE genes, 69 upregulated and 67 downregulated in *MYCN*-T58A (Fig. 6H). The sample correlation matrix shows large overlap among the samples suggesting overall similarity of expression profile of the samples which is also reflected in small number of differentially expressed genes within groups but also between the T58A DMC4-6 and “old” DMC1-3 (Fig. 6I). Among the top 20 DE genes, genes including *HINTW* located on the female W sex determining chromosome stood out. The genes have sex-specific expression and were removed from the analysis. The top 20 genes without W-chromosome- and uncharacterized genes are shown in Fig. 6J. No enriched GO or KEGG pathways were identified in the data with comparison to the *MYCN* cells indicating that the T58A cells are relatively similar to both the “young” and the “old” DMC MYCN expressing cells.

## Discussion

In this work, we have studied in vivo-induced retinoblastoma tumour cell lines with over-expression of *MYCN*. The tumours were generated in normal chicken embryonic retina and represent an early established type of *RB1* proficient *MYCN*^A^ retinoblastoma cancer [7]. The expression profiles show that the tumour cells have a clear cPR signature together with a wide spectrum of *MYCN*-induced genes. This profile is consistent with an origin of the tumour in the cPR lineage, which is similar to the cell-of-origin for the *RB1*-deficient cancers [15]. The profile revealed that the expression of several of the activating *E2F* gene family members were upregulated. The activity of E2fs is directly regulated by the Rb protein and in spite of *RB1* proficiency our results suggest that the increased levels of E2f contribute to a dysfunctional cell cycle regulation and hence to the cancer phenotype. Our results also suggest that the cells are to some degree resistant to p53 activation. Such resistance is also consistent with the properties and cellular origin of a cell in the cPR lineage.

The cell lines were established directly after the initial signs of tumour formation in the E14 embryonic retina and thus represents an early pristine form of this type of retinoblastoma. Previous research has suggested the existence of two types of retinoblastomas based on their genetics and pathology. Type 1 tumours harbour few genetic alterations other than the initiating *RB1*-inactivating mutations and corresponds to differentiated tumours expressing mature cone markers. By contrast, type 2 tumours harbour frequently recurrent genetic alterations including *MYCN*-amplifications. They express markers of less differentiated cones together with other neuronal and ganglion cell markers, with marked tumour heterogeneity. The undifferentiated phenotype in type 2 is associated with stemness features including low immune and interferon response, *E2F* and *MYCN* activation and a high propensity for metastasis [5]. The tumour cells established in this work represent a tumour with *MYCN* over-expression but without *RB1* inactivation or other genetic lesions. The phenotype is anaplastic and aggressive as shown by optic nerve infiltration and extraocular growth [7]. The clear cPR signature of phototransduction gene expression is accompanied with genes for cPR progenitors such as *RXRG* and *THRB*, indicative of progenitors or immature cPRs reflecting the corresponding early events during carcinogenesis. The majority of the top enriched, down regulated GO terms are related to neuronal development. None of the DMC cells display any overt ganglion cell markers as several of the type 2 cancers do [5], or that is seen in tumours after MYCN expression in foetal human retina transduced in vitro and xenografted to mice [8]. This may be due to the pristine state of the chicken “young” DMC cells taken only a day after tumour formation and few days in culture. The cells have then not been under influence of *MYCN* for the extended period after transformation which is the case for patient-derived or xenografted experimental tumours. The “old” DMC cells that had been in culture for more than 200 days and the “young” DMC cells were very similar with relatively few DE genes and without any significant enriched GO-terms or pathways. However, when looking at individual DE genes, a trend with lower expression of cPR genes such as *ARR3, RXRG, THRB* as well as several phototransduction genes, was seen in the “old” (MYCN and T58A) cells. This would suggest that prolonged actions of *MYCN* in the tumour cells may facilitate dedifferentiation of the original cPR phenotype. Genes related to early retinal development, such as *IRX3*, was also higher in the “old” cells. Species differences may also contribute to the differences and the stage of cPR development in relation to the effective time of transformation. The chicken retina develops fast and cPR are early formed which may render a relatively advanced stage of cPR at transformation.

The DMC cells can be established with high frequency (100% of successful *in ovo* electroporations) from dissected embryonic retinal tumours and the *MYCN* tumour cells grow in suspension with concomitant cell death similar to patient derived *MYCN*-retinoblastoma lines [36, 42]. The transcriptional profile of the chicken tumour cells has a clear cone photoreceptor signature in addition to broader signatures with increased biosynthesis and cell cycle regulation while that of neuronal development, differentiation and signalling is decreased, compared to E14 retina. Such effects on the expression profile can be expected from the impact of *MYCN* over-expression. *MYC* family members are known to drive proliferation and stem cell-ness at the expense of a differentiated cell phenotype with upregulation of cell cycle regulators such as CDKs and E2fs [21]. Upregulation of these genes has also been found in different retinoblastoma cell lines [53]. While the *E2F* family members were upregulated, the *RB1* expression was not changed in the DMC-cells and we confirmed presence of Rb-protein using immunocytochemistry for Rb IR as well as for phospho-S608-Rb. Phosphorylation of Rb on S608 was blocked by the Cdk4/6 inhibitor Palbociclib (Fig. 1). Furthermore, Palbociclib had a significant but limited effect on proliferation and the cell cycle of DMC cells, leading to more cells in G1-phase, which is significative for increased number of cells in G1-arrest as a result of reduced phosphorylation of Rb. Such effects corroborate that the DMC cells have functional Rb protein, which is phosphorylated by Cdk4/6 and that the cells in fact are *RB1* proficient. *RB1* mutations and gene loss have been shown to induce resistance to Palbociclib in breast cancer cells [54]. DMC cells do not display resistance to Palbociclib, which support that the DMC cells are *RB1* proficient.

Upon phosphorylation of Rb, E2f1 is disinhibited and promotes transcription of factors that transit cells into S-phase. As long as Rb is unphosphorylated and the pool of Rb is sufficient to inhibit the E2fs, the cell cycle will be regulated. With a large pool of E2fs, as seen in the DMC-tumour cells, Rb will not be able to fully inhibit E2f in the cell, leading to uncontrolled proliferation. A block downstream of E2f will, however, reduce proliferation and we showed that HLM006474 led to decreased proliferation and increased G1-phase arrest. Addition of increasing concentrations of Palbociclib with HLM006474 did not increase the effect while when increasing the concentration of HLM006474 the inhibitory effects were significantly higher. Interestingly, prolonged treatment with HLM006474 induced cell cycle arrest and cell death (Fig. 5L-M), which is consistent with that E2fs and not Rb constitutes the rate limiting step in the regulatory pathway of the DMC cells.

As already introduced, the tumour cells are derived from a retinal progenitor that do not exhibit the extent of developmental cell death as seen in other retinal cell types [13, 14], it withstands DNA-damage and escapes cell cycle arrest and apoptosis [16–19]. Such resistance against p53-induced death was suggested to be a result of insensitivity to modulation of the p53 activity by the MDM2 inhibitor Nutlin-3a, the p53 coactivator Zac1 and p53 inhibitor pifithrin-a [17, 19]. Insensitivity to Nutlin-3a and pifithrin-a was replicated in the DMC cells implying that such p53 resistance is remaining after transformation (Fig. 4). Furthermore, an aberrant p53 pathway regulation was identified as top-ranked in the enriched activated pathways (Fig. 3I) and together this implies that p53 is dysregulated, which may contribute to the tumorigenic phenotype. An intrinsic p53 insensitivity, rather than effects of *TP53* mutations, is consistent with that silencing mutations in *TP53* are seldom found in retinoblastoma [55]. Altered p53 functions in these cells is also consistent with the absence of upregulation of *CDKN1A* mRNA after Nutlin-3a or pifithrin-a treatments (Fig. 4I). However, the intrinsic p53 insensitivity seams not to give cells resistance to apoptosis as shown by *CDKN1A* upregulation, cell cycle arrest and extensive apoptosis after treatment with HLM006474 (Fig. 5M and N). It is not clear where in the regulatory pathways the insensititvity resides. We do not find upregulation of *MDM2* mRNA but robust expression of *CDKN2A*. Human cPR precursors express high levels of *MDM2* and patient-derived retinoblastoma cells require functional *MDM2* for survival which also suppresses *CDKN2A*/p14ARF-induced apoptosis in cultured human retinoblastoma cells [15]. Furthermore, there is an extensive literature on blocking Mdm2 and its effects on retinoblastoma (reviewed in Laurie et al. 2007) [56]. *MDM2* has been shown to promote proliferation in both neuroblastoma and retinoblastoma cells through p53-independent regulation of *MYCN* [57, 58]. High transgene *hMYCN* expression is likely making this pathway redundant. Noteworthy, we see *CDKN1A* upregulation after blocking E2f, which also is consistent with a potential p53-independent regulatory pathway (Fig. 5F).

In conclusion, the results from this work implies that *MYCN*-induced E2f-expression, contributes to a dysfunctional cell cycle regulation. In spite of *RB1*-proficiency, the increased levels of E2f cause a cancer phenotype that resembles that of a *RB1*-deficient retinoblastoma. The expression profiles show that these tumour cells have a clear cone photoreceptor signature together with a wide spectrum of MYCN-induced genes. Such profile is consistent with an origin of the tumour in the cone progenitor lineage and is similar to the cell-of-origin for *RB1*-deficient retinoblastomas. Our results also suggest that the cells are to some degree insensitive to p53 activation, which is consistent with the properties and cellular origin of a cell in the cone progenitor lineage. The results, based on the chicken as a model show that the mechanism for retinoblastoma is among several evolutionary conserved pathogenetic mechanisms for human disease [59]. The implications of these results are that targeting the Cdk4/6 - E2f signalling pathway may be a relevant and complementary regime for retinoblastoma with *MYCN*^*A*^s. In *RB1* proficient tumours with elevated *E2F* expression, it may not be sufficient to target Cdk4/6 with Palbociclib but also target the E2fs. *MYCN* mutations is a known aggravating factor in paediatric cancers and defines severe subtypes of medulloblastoma and neuroblastoma. *MYCN* is therefore an attractive drug target, however, direct targeting of MYCN has proven difficult [60]. Targeting key *MYCN* down-stream effectors such as elevated E2fs may be a feasible way to treat those cancers including retinoblastoma with *MYCN*^*A*^.

### Supplementary information

Below is the link to the electronic supplementary material.

ESM 1 (PDF 141 KB)

ESM 2 (PDF 1.61 MB)

ESM 3 (PDF 654 KB)

ESM 4 (PDF 104 KB)

ESM 5 (PDF 121 KB)

ESM 6 (XLSX 0.99 MB)

ESM 7 (XLSX 14.9 KB)

ESM 8 (XLSX 13.3 KB)

ESM 9 (XLSX 14.8 KB)

ESM 10 (XLSX 14.8 KB)

## Data Availability

All data generated or analysed during this study are included in the published article and its Supplementary information files. Raw data is available under accession GSE226458 in the NCBI GEO data repository or from the corresponding author upon reasonable request.
