# Supplementary material for: Inhibition of high level E2F in a RB1 proficient MYCN overexpressing chicken retinoblastoma model normalizes neoplastic behaviour
Source: Cell Oncol (Dordr). 2023 Aug 22;47(1):209–27. doi: 10.1007/s13402-023-00863-0 (PMC10899388; doi:10.1007/s13402-023-00863-0)

Supplementary figure S1

Cellular Oncology

Inhibition of high level *E2F* in a *RB1* proficient *MYCN* overexpressing retinoblastoma model normalizes neoplastic behaviour

Hanzhao Zhang (1), Dardan Konjusha (1), Nima Rafati (2,3), Tatsiana Tararuk (1) and Finn Hallböök (1)*

Affiliations:

1. Department of Immunology, Genetics and Pathology, Uppsala University,
2. National Bioinformatics Infrastructure Sweden, Science for Life Laboratory, Uppsala University
3. Department of Medical Biochemistry and Microbiology, Uppsala University, Uppsala, Sweden

* Corresponding author:
Finn Hallböök
Department of Immunology, Genetics and Pathology
Rudbeck laboratory, Uppsala University
751 85 Uppsala Sweden
Finn.Hallbook@igp.uu.se

Fig. S1. Gene set enrichment analysis (GSEA) of GO terms of comparison between DMC and E14 retina

(A-B) Top 10 downregulated and upregulated GO terms in cellular components (CC) from GSEA.

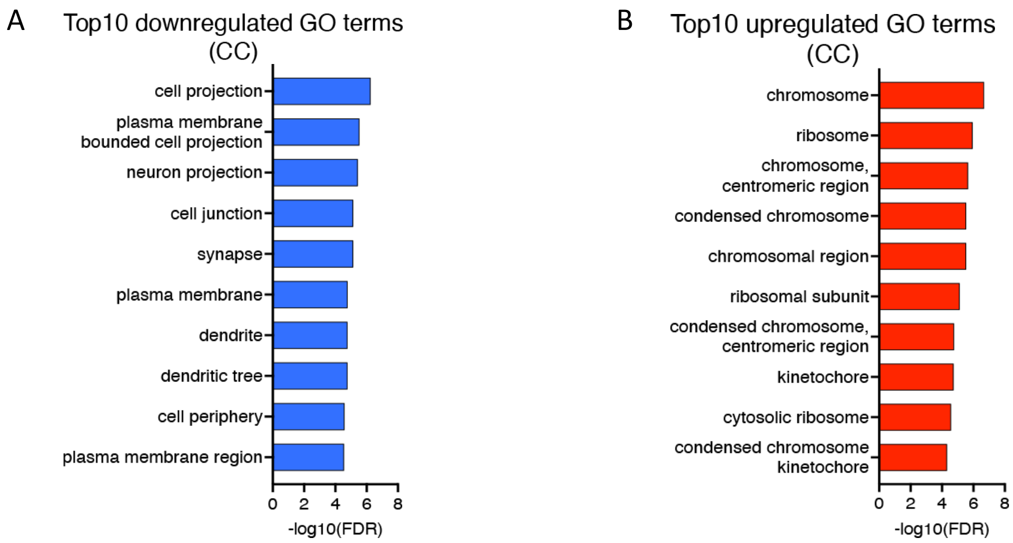

Supplement: Supplementary file 1 — (PDF 141 KB) [file 13402_2023_863_MOESM1_ESM.pdf]
