# Supplementary material for: Inhibition of high level E2F in a RB1 proficient MYCN overexpressing chicken retinoblastoma model normalizes neoplastic behaviour
Source: Cell Oncol (Dordr). 2023 Aug 22;47(1):209–27. doi: 10.1007/s13402-023-00863-0 (PMC10899388; doi:10.1007/s13402-023-00863-0)

Supplementary figure S2

Cellular Oncology

Inhibition of high level *E2F* in a *RB1* proficient *MYCN* overexpressing chicken retinoblastoma model normalizes neoplastic behaviour

Hanzhao Zhang (1), Dardan Konjusha (1), Nima Rafati (2,3), Tatsiana Tararuk (1) and Finn Hallböök (1)*

Affiliations:

1. Department of Immunology, Genetics and Pathology, Uppsala University,
2. National Bioinformatics Infrastructure Sweden, Science for Life Laboratory, Uppsala University
3. Department of Medical Biochemistry and Microbiology, Uppsala University, Uppsala, Sweden

* Corresponding author:
Finn Hallböök
Department of Immunology, Genetics and Pathology
Rudbeck laboratory, Uppsala University
751 85 Uppsala Sweden
Finn.Hallbook@igp.uu.se

Fig. S2. Expression profile from two patient-derived MYCNA tumours

Enriched GO terms based on DE genes from two patient-derived *MYCN*[A] tumours. **A** Top 20 enriched GO terms of downregulated DE genes of public available human dataset (https://www.ncbi.nlm.nih.gov/geo/, GSE161449). The comparison is patient-derived *MYCN*[A] *RB1* proficient retinoblastoma cell lines versus the same cell line with *MYCNOS1* silencing. **B** Top 20 enriched GO terms of upregulated DE genes of public available human dataset (https://www.biosino.org/node/index, OEP000103). The comparison is *MYCN*[A] *RB1* deficient retinoblastoma tumour versus para tumour tissue. **C** Top 20 enriched GO terms of downregulated DE genes of public available human dataset (https://www.biosino.org/node/index, OEP000103). The comparison is *MYCN*[A] *RB1* deficient retinoblastoma tumour versus para tumour tissue.

A Top 20 enriched GO terms of downregulated DE genes (GSE161449)

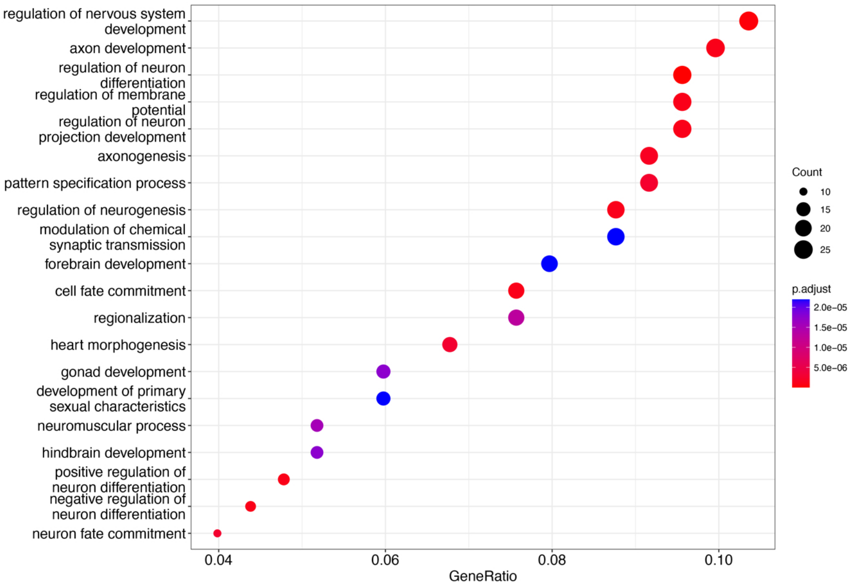

B Top 20 enriched GO terms of upregulated DE genes (OEP000103)

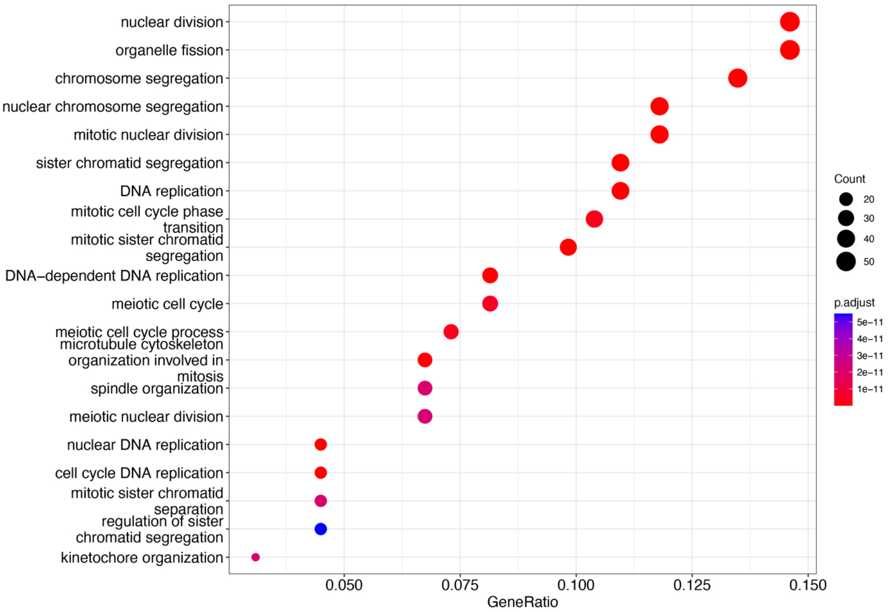

C Top 20 enriched GO terms of downregulated DE genes (OEP000103)

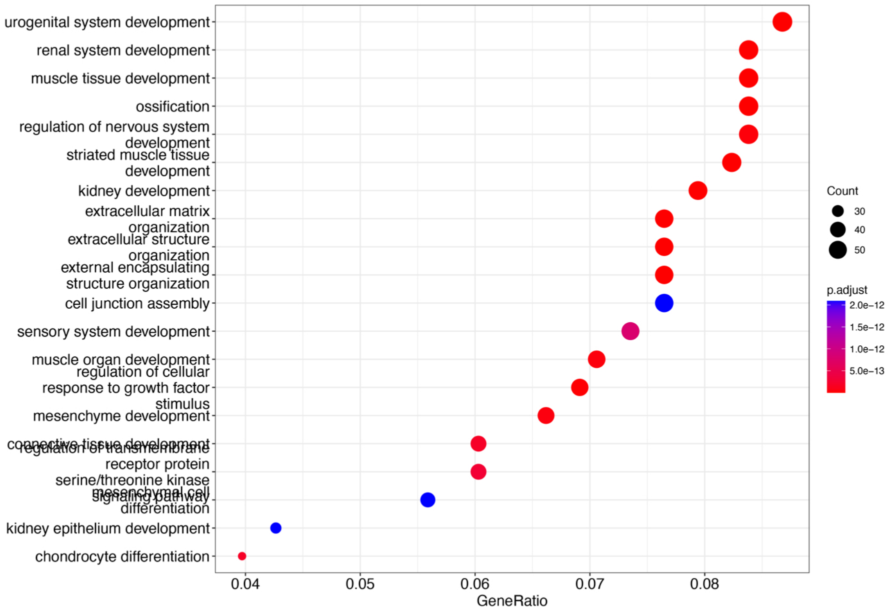

Supplement: Supplementary file 2 — (PDF 1.61 MB) [file 13402_2023_863_MOESM2_ESM.pdf]
