# Supplementary material for: Inhibition of high level E2F in a RB1 proficient MYCN overexpressing chicken retinoblastoma model normalizes neoplastic behaviour
Source: Cell Oncol (Dordr). 2023 Aug 22;47(1):209–27. doi: 10.1007/s13402-023-00863-0 (PMC10899388; doi:10.1007/s13402-023-00863-0)
Supplement: Supplementary file 3 — (PDF 654 KB) [file 13402_2023_863_MOESM3_ESM.pdf]

Supplementary figure S3

Cellular Oncology

Inhibition of high level *E2F* in a *RB1* proficient *MYCN* overexpressing retinoblastoma model normalizes neoplastic behaviour

Hanzhao Zhang (1), Dardan Konjusha (1), Nima Rafati (2,3), Tatsiana Tararuk (1) and Finn Hallböök (1)*

Affiliations:

1. Department of Immunology, Genetics and Pathology, Uppsala University,
2. National Bioinformatics Infrastructure Sweden, Science for Life Laboratory, Uppsala University
3. Department of Medical Biochemistry and Microbiology, Uppsala University, Uppsala, Sweden

* Corresponding author:
Finn Hallböök
Department of Immunology, Genetics and Pathology
Rudbeck laboratory, Uppsala University
751 85 Uppsala Sweden
Finn.Hallbook@igp.uu.se

Fig. S3. Relative mRNA levels and normalized counts of hMYCN-GFP, MKI67, E2F1, E2F3, RB1 and PRDM13.

To verify the RNA sequencing analyses we performed qRT-PCR analysis of one sample of the "young" MYCN cells (DMC8) and E14 retina. The result was compared to that of the RNA seq. analysis. The qRT-PCR result for each gene was normalized to the average level of three house-keeping genes: b-actin, GAPDH and TBP. **(A)** Bar graphs showing mRNA levels relative to the average house-keeping gene level. Mean+/- SD of the technical replicates of one analysed sample, n=1. **(B)** Bar graphs showing the mean normalized counts from three samples of "young" MYCN cells (DMC8) vs E14 retina. Mean +/- SD, n=3.

A

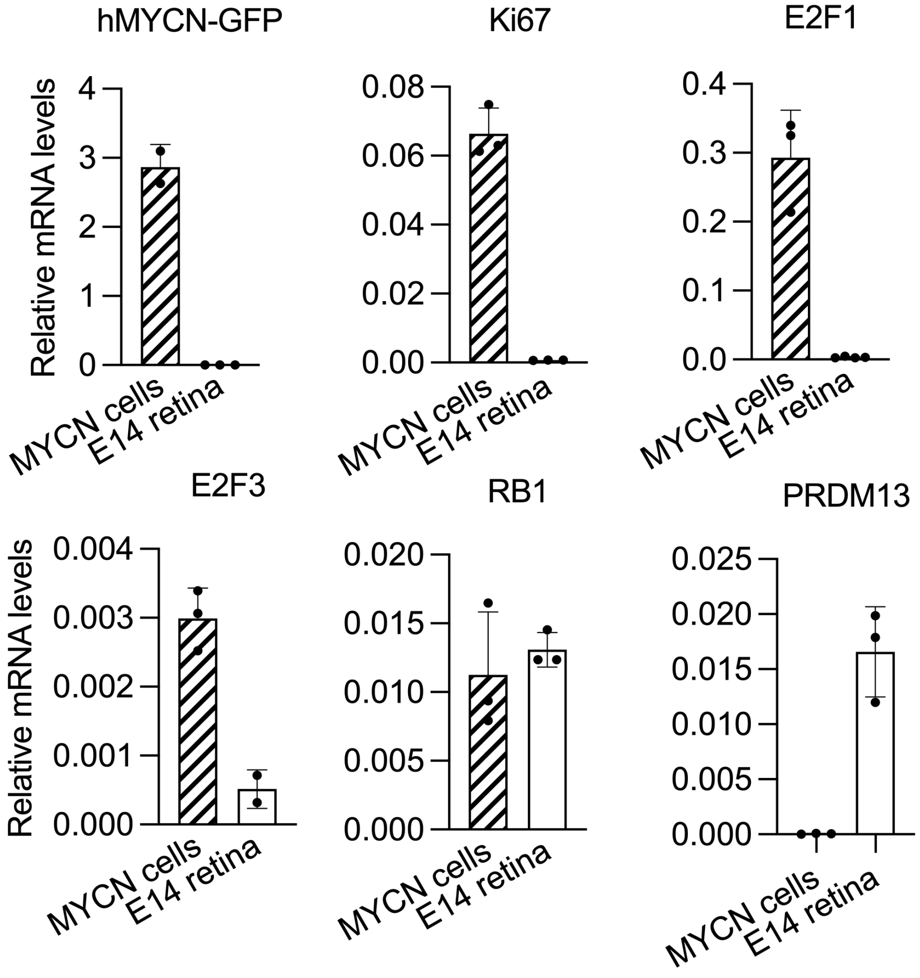

B

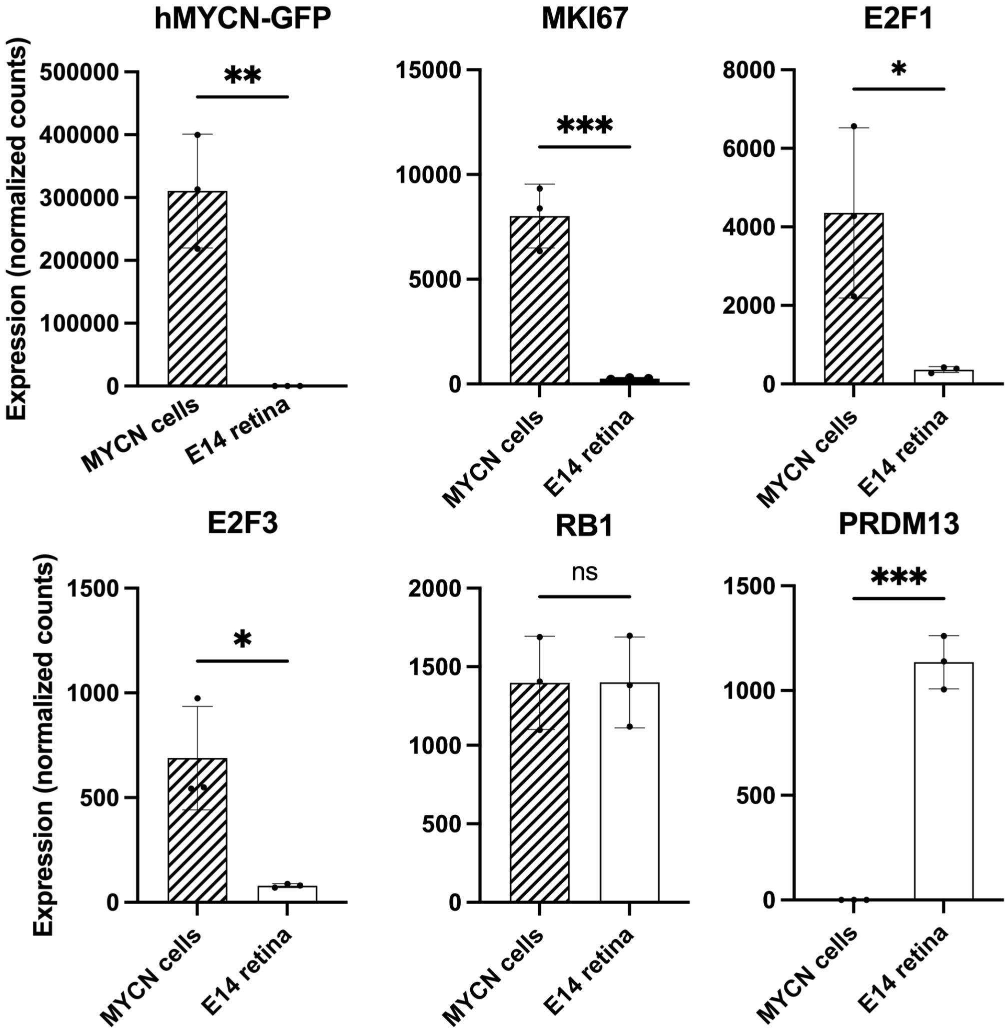
hMYCN-GFP
MKI67
E2F1
E2F3
RB1
PRDM13
Expression (normalized counts)
500000
400000
300000
200000
100000
0
15000
10000
5000
8000
6000
4000
2000
1500
1000
500
**
***
*
ns
MYCN cells
E14 retina
