# Supplementary material for: Inhibition of high level E2F in a RB1 proficient MYCN overexpressing chicken retinoblastoma model normalizes neoplastic behaviour
Source: Cell Oncol (Dordr). 2023 Aug 22;47(1):209–27. doi: 10.1007/s13402-023-00863-0 (PMC10899388; doi:10.1007/s13402-023-00863-0)
Supplement: Supplementary file 4 — (PDF 104 KB) [file 13402_2023_863_MOESM4_ESM.pdf]

Supplementary Table S1: List of antibodies used in this study

Cellular Oncology

Inhibition of high level *E2F* in a *RB1* proficient *MYCN* overexpressing chicken retinoblastoma model normalizes neoplastic behaviour

Hanzhao Zhang (1), Dardan Konjusha (1), Nima Rafati (2,3), Tatsiana Tararuk (1) and Finn Hallböök (1)*

Affiliations:

1. Department of Immunology, Genetics and Pathology, Uppsala University,
2. National Bioinformatics Infrastructure Sweden, Science for Life Laboratory, Uppsala University
3. Department of Medical Biochemistry and Microbiology, Uppsala University, Uppsala, Sweden

* Corresponding author:
Finn Hallböök
Department of Immunology, Genetics and Pathology
Rudbeck laboratory, Uppsala University
751 85 Uppsala Sweden
Finn.Hallbook@igp.uu.se

Supplementary Table S1: List of antibodies used in this study

Antibody	Host	Dilution	Company	Catalogue number
Human MYCN	mouse	1:250	Abcam	ab16898
Actin	rabbit	1:3000	SigmaAldrich	A2066
Rb	rabbit	1:1000	Abcam	ab39690
Rb (P-ser608)	rabbit	1:1000	Abcam	ab60025
Visinin	mouse	1:1000	DSHB*	7G4
Cleaved Caspase-3	rabbit	1:1000	Cell Signaling	9661
E2F1**	rabbit	1:500	LSBio	LS-C102852
E2F1**	mouse	1:500	Santa Cruz	sc-251

*DSHB: Developmental Studies Hybridoma Bank

**: Antibody not working
