# Supplementary material for: Inhibition of high level E2F in a RB1 proficient MYCN overexpressing chicken retinoblastoma model normalizes neoplastic behaviour
Source: Cell Oncol (Dordr). 2023 Aug 22;47(1):209–27. doi: 10.1007/s13402-023-00863-0 (PMC10899388; doi:10.1007/s13402-023-00863-0)
Supplement: Supplementary file 5 — (PDF 121 KB) [file 13402_2023_863_MOESM5_ESM.pdf]

**Supplementary Table S2:** List of primers for qRT-PCR

*Cellular Onncology*

# Inhibition of high level *E2F* expression in a *RB1* proficient *MYCN* overexpressing retinoblastoma model normalizes the neoplastic behaviour

Hanzhao Zhang (1), Dardan Konjusha (1), Nima Rafati (2,3), Tatsiana Tararuk (1) and Finn Hallböök (1)*

## Affiliations:

1. Department of Immunology, Genetics and Pathology, Uppsala University,
2. National Bioinformatics Infrastructure Sweden, Science for Life Laboratory, Uppsala University
3. Department of Medical Biochemistry and Microbiology, Uppsala University, Uppsala, Sweden

* Corresponding author:
Finn Hallböök
Department of Immunology, Genetics and Pathology
Rudbeck laboratory, Uppsala University
751 85 Uppsala Sweden
Finn.Hallbook@igp.uu.se

**Supplementary Table S2:** List of primers for qRT-PCR

| Gene | Forward 5'-3' | Reverse 5'-3' | Source |
|---|---|---|---|
| β-actin | aggtcatcaccattggcaatg | cccaagaaagatggctggaa | PMID:25483080 |
| GAPDH | cctgacctgccgtctagaaaaa | tgtcgctgttgaagtcagagga | |
| p21 | caatgccgagtctgtagttccc | cttccagtcctcctcagtccctt | |
| TBP | tagcccgatgatgccgtat | gttccctgtgtcgcttgc | PMID:25483080 |
| MYCN (Human) | ttgtacaaaaaagcaggctccg | agcgagtcaaactcgaggtctg | from vector |
| E2F3 | agaaagcggtcaatcagtacct | tggacttcgtagtgcagctct | PMID:34062439 |
| PRDM13 | acccaagaaaaaggtgcgga | tatccactccaaagcggagc | |
| Ki67 | tggtgatttgttacctcagcct | gtggcgagtttccaaatgg | XM_015289038.1 |
| RB1 | aacagcgagagccacgtaaa | tatttgcctccatctgcctc | |
